# Automated classification of clinical diagnoses in electronic health records using transformer

**DOI:** 10.1371/journal.pone.0329963

**Published:** 2025-09-11

**Authors:** Lixia Dai, Hang Xu, Yugui Zhang

**Affiliations:** 1 Antai College of Economics and Management, School of Shanghai Jiao Tong University, Shanghai, China; 2 Vanke School of Public Health, Tsinghua University, Beijing, China; 3 Institute of Semiconductors, Chinese Academy of Sciences, Beijing, China; Universita degli Studi di Siena, ITALY

## Abstract

The automated classification of clinical diagnoses in electronic health records (EHRs) is critical for enhancing clinical decision-making and enabling large-scale medical research, yet existing methods struggle with heterogeneous data structures and limited annotated datasets. Current approaches fail to adequately address the dual challenges of extracting contextual medical semantics from unstructured clinical narratives while maintaining generalizability across institutions with divergent documentation practices. This study proposes a novel framework integrating three core components: a Transformer-based architecture for hierarchical feature extraction from clinical text, a multi-task learning paradigm leveraging diagnostic interdependencies, and transfer learning initialization using pretrained medical language models. Evaluation on the MIMIC-III dataset demonstrates state-of-the-art performance with 89.2% accuracy and 87.6% F1-score, outperforming conventional CNN-RNN hybrids by 8.0% in recall and showing 4.9-6.2% improvements over ablated configurations in critical metrics. The results establish that synergistic integration of contextual attention mechanisms, cross-task knowledge sharing, and medical domain adaptation effectively addresses EHR heterogeneity while reducing reliance on institution-specific annotations, providing a robust foundation for clinical decision support systems that balance accuracy with real-world implementability across diverse healthcare environments.

## 1 Introduction

The automated classification of clinical diagnoses in electronic health records (EHRs) holds significant importance for modern healthcare systems [1], as it can substantially enhance clinical decision-making [2], streamline administrative workflows [3,4], and improve patient outcomes. With the exponential growth of digital health data, manually processing and categorizing vast amounts of clinical information has become increasingly inefficient and error-prone. Automated classification systems can alleviate this burden by rapidly and accurately organizing diagnoses into structured formats, enabling healthcare providers to access relevant patient information more efficiently. This capability is particularly valuable in time-sensitive scenarios, such as emergency care or chronic disease management [5], where timely and precise diagnosis categorization can directly influence treatment efficacy. Furthermore, standardized and machine-readable diagnostic data facilitate large-scale epidemiological studies [6], allowing researchers to identify disease patterns [7], monitor public health trends [8], and evaluate treatment effectiveness across diverse populations [9]. From an operational perspective, automated classification reduces documentation errors, minimizes redundant data entry, and optimizes billing and insurance claim processing, thereby reducing healthcare costs and administrative overhead. Additionally, integrating such systems into clinical workflows can support predictive analytics, enabling early detection of potential health risks and personalized treatment recommendations. Beyond immediate clinical applications, structured and classified EHR data enhance interoperability between different healthcare systems, fostering better care coordination among multiple providers and institutions. Despite these advantages, challenges such as data heterogeneity, privacy concerns, and model interpretability remain, underscoring the need for robust and ethical AI solutions in healthcare. Overall, the automated classification of clinical diagnoses represents a critical step toward data-driven medicine, with the potential to transform healthcare delivery by improving the accuracyracy, efficiency, and accessibility of medical information.

Deep learning has revolutionized the automated classification of clinical diagnoses in electronic health records (EHRs) by overcoming key limitations of traditional machine learning approaches. Conventional methods often required extensive feature engineering and struggled with the high dimensionality [10], temporal dependencies [11], and unstructured nature of EHR data. Deep neural networks, with their hierarchical feature learning capabilities, can automatically extract meaningful patterns from raw EHR data without manual feature selection [12]. Architectures such as convolutional neural networks (CNNs) [13] have proven effective at processing clinical notes through text embedding layers, while recurrent neural networks (RNNs) [14] and their variants like long short-term memory (LSTM) networks [15] excel at modeling temporal relationships in patient histories. More recently, transformer-based models leveraging self-attention mechanisms have demonstrated state-of-the-art performance by capturing long-range dependencies in clinical text and enabling parallel processing of sequential data [16]. These models can integrate multimodal EHR components including structured diagnostic codes, unstructured clinical notes, laboratory results, and medication records into unified classification frameworks. The application of transfer learning, particularly through pretrained language models fine-tuned on medical corpora, has further enhanced performance while reducing data requirements. However, challenges remain in handling data heterogeneity across institutions, addressing class imbalance in rare diagnoses, and ensuring model interpretability for clinical adoption.

Despite their promise, several practical considerations must be addressed when implementing deep learning for diagnostic classification in clinical settings. The black-box nature of deep neural networks raises concerns about explainability, prompting the development of attention mechanisms and saliency mapping techniques to provide clinical rationale for predictions. Data quality issues including missing values, inconsistent documentation practices, and labeling noise require specialized preprocessing and robust training strategies. Privacy preservation through techniques like federated learning has become increasingly important for multi-institutional collaborations while complying with healthcare regulations. Computational resource requirements for training large models and latency constraints for real-time clinical deployment present additional engineering challenges. Future directions include the development of hybrid models combining deep learning with knowledge graphs or ontologies, integration of patient-specific contextual information, and the creation of standardized benchmarks for fair performance comparison. As these technologies mature, their successful translation to clinical practice will depend not only on algorithmic advances but also on seamless integration with existing clinical workflows, rigorous validation across diverse patient populations, and the establishment of trust among healthcare providers through transparent and clinically meaningful model outputs.

Deep Learning EHR Classification The integration of deep learning (DL) techniques into electronic health record (EHR) classification has emerged as a transformative approach in healthcare [17], which introduces HORDE, a unified graph representation learning framework designed to embed heterogeneous medical entities into a harmonized space. This framework addresses the inconsistencies often found in structured codes by incorporating unstructured clinical data, such as clinical notes, thereby enhancing the robustness of downstream analyses. The ability to fuse detailed information from diverse sources is crucial for improving classification accuracy in EHR systems. Goodrum [18] further explore the classification of EHR documents by developing a system that categorizes scanned documents into clinically relevant and non-clinically relevant categories. Their work emphasizes the importance of document classification in managing the vast array of information contained within EHRs, which includes identification cards, radiology reports, and clinical correspondence. This classification is essential for streamlining clinical workflows and ensuring that healthcare providers can access pertinent information efficiently. Minaee [19] provide a comprehensive review of over 150 deep learning models for text classification, underscoring the superiority of DL approaches over traditional machine learning methods in various tasks, including those relevant to EHRs. Their findings suggest that deep learning models can significantly enhance the performance of text classification tasks, which is particularly relevant for extracting meaningful insights from unstructured clinical data.

In the context of specific medical conditions, Mitra [20] investigate the classification of relations between bleeding events and other medical concepts using deep learning systems. Their empirical study highlights the potential of DL to uncover complex relationships within EHR data, which can inform clinical decision-making and improve patient outcomes. Similarly, Sheu [21] apply deep learning to phenotype antidepressant treatment responses, demonstrating the capability of AI methods to tackle complex classification problems in mental health.

The integration of natural language processing (NLP) with deep learning is another significant trend in EHR classification. Liu [22] illustrate how the NLP2FHIR representation can be utilized for clinical phenotyping, showcasing a case study on obesity datasets. This approach not only enhances the classification of clinical data but also facilitates the normalization of unstructured data into a format suitable for deep learning models. Moreover, the work of Han [23] focuses on the extraction of social determinants of health (SDOH) from unstructured EHRs using advanced deep learning techniques. Their systematic approach to creating a comprehensive set of SDOH based on standard ontologies demonstrates the potential for deep learning to automate the extraction of critical social factors that influence health outcomes.

The predictive capabilities of deep learning in EHR classification are further exemplified by McGilvray [24], who develop models to predict death or severe decompensation in heart failure patients. Their study emphasizes the importance of leveraging commonly available EHR variables to assist clinicians in timely decision-making, thereby improving patient care. Recent advancements also include the exploration of multimodal data in EHR classification. Zhang [25] propose using multimodal longitudinal EHR data to predict multiple sclerosis disease severity, achieving significant improvements in predictive accuracy. This highlights the potential of integrating diverse data types to enhance classification outcomes. Despite these advancements, challenges remain in the field of deep learning EHR classification. Issues such as data imbalance and missing values can hinder model performance. Chang [26] address these challenges by introducing a meta-learning approach to manage noise in EHR datasets, which is crucial for achieving reliable classification results. In conclusion, the application of deep learning to EHR classification is a rapidly evolving field that holds significant promise for enhancing healthcare delivery. The integration of diverse data sources, advanced NLP techniques, and multimodal approaches are paving the way for more accurate and efficient classification systems. However, ongoing research is needed to address the challenges of data quality, model interpretability, and the ethical implications of AI in healthcare. As the field progresses, the potential for deep learning to transform EHR classification and improve patient outcomes continues to expand.

This study proposes a novel deep learning framework for automated clinical diagnosis classification from electronic health records, integrating three key technical components in a sequential pipeline. The first module employs a Transformer-based architecture to process unstructured clinical text data, generating dense vector representations that capture critical medical semantics through self-attention mechanisms. These embeddings preserve contextual relationships between medical concepts while addressing the challenge of information extraction from heterogeneous clinical narratives. The second module implements a multi-task learning (MTL) paradigm that simultaneously processes the Transformer-generated embeddings alongside structured patient metadata, enabling joint optimization of multiple clinically relevant prediction tasks through shared parameter learning. This design capitalizes on task interdependencies to enhance diagnostic classification accuracy while reducing model complexity compared to training separate task-specific models. The final component incorporates transfer learning methodology, initializing the Transformer module with weights pretrained on external medical corpora before fine-tuning on target EHR datasets. This approach addresses data scarcity constraints by transferring generalized medical knowledge from source domains while adapting to institution-specific documentation patterns. The integrated architecture strategically combines these technologies to overcome fundamental challenges in clinical NLP: Transformer layers handle textual complexity through hierarchical feature learning, MTL improves predictive performance via knowledge sharing across related medical tasks, and transfer learning boosts generalization while minimizing training resource requirements. The design particularly emphasizes clinical applicability by maintaining model interpretability through attention visualization and supporting incremental updates as new patient data becomes available. This comprehensive approach aims to establish a robust foundation for automated diagnostic support systems that can scale across diverse healthcare settings while accommodating the dynamic nature of medical knowledge evolution.

The contributions of this paper are as follows:

1. The integration of Transformer-based semantic modeling with multi-task learning enables simultaneous optimization of diagnostic classification and related clinical predictions through shared representation learning.
2. A novel hybrid architecture combines pretrained language model knowledge with domain-specific fine-tuning, addressing data scarcity while preserving medical context across institutions.
3. The framework implements hierarchical feature extraction by processing both unstructured clinical narratives and structured metadata in a unified diagnostic decision pipeline.

The paper systematically presents an automated diagnosis classification framework through seven organized sections. The Introduction establishes the clinical significance of EHR processing and identifies limitations in current methods, followed by Related Work reviewing three key areas: healthcare AI fundamentals, EHR analysis techniques, and clinical text mining advancements. The Method section details the proposed architecture through technical subsections: an Overview explaining the integrated transformer-MTL-transfer learning pipeline, Data Preprocessing describing specialized clinical text normalization, and Implementation specifying model configurations. Subsequent sections present Experimental Setup comparing benchmark datasets and evaluation metrics, Results showcasing performance against baselines with ablation studies, and Discussion interpreting clinical applicability while addressing ethical considerations. The conclusion synthesizes technical contributions and healthcare impacts, with appendices providing implementation specifics.

## 2 Related work

### 2.1 Healthcare AI

Healthcare AI represents a transformative approach in modern medicine that leverages artificial intelligence to analyze complex medical data and improve healthcare delivery [27]. This interdisciplinary field combines machine learning, natural language processing, and computer vision to extract meaningful insights from diverse healthcare data sources including electronic health records, medical images, and genomic sequences. The fundamental principle involves training algorithms on large medical datasets to recognize patterns, predict outcomes, and assist clinical decision-making, while continuously improving through iterative learning processes. Various architectures such as deep neural networks [28], random forests [29], and support vector machines [30] are adapted to handle the unique challenges of healthcare data including its heterogeneity, temporal nature, and privacy requirements.

The technology can be broadly categorized into diagnostic AI [31], predictive analytics [32], and operational optimization systems [33]. Diagnostic tools [34] primarily focus on image recognition and clinical text interpretation, while predictive models forecast disease progression and treatment responses [35,36]. Operational applications streamline hospital workflows and resource allocation. Recent advances incorporate explainable AI techniques [37] to enhance model transparency and hybrid approaches that combine multiple data modalities. Emerging subfields include federated learning for privacy-preserving multi-institutional collaborations and reinforcement learning for personalized treatment optimization.

Key advantages of healthcare AI include its ability to process vast amounts of data beyond human capacity, identify subtle correlations invisible to clinicians, and provide real-time decision support. These systems demonstrate particular strength in repetitive pattern recognition tasks, reducing diagnostic errors and variability in clinical interpretations. AI applications can operate continuously without fatigue, enabling round-the-clock patient monitoring and early warning systems. The technology also facilitates personalized medicine by analyzing individual patient characteristics against population-level data, potentially improving treatment efficacy while reducing adverse effects.

Application scenarios span the entire healthcare continuum from prevention to palliative care. In clinical settings, AI assists with medical imaging interpretation, clinical documentation, and risk stratification. Pharmaceutical companies employ AI for drug discovery and clinical trial optimization. Public health organizations utilize predictive models for disease surveillance and outbreak forecasting. Patient-facing applications include virtual health assistants and remote monitoring tools. Administrative functions benefit from AI-powered billing automation, fraud detection, and resource scheduling systems.

### 2.2 EHR data analysis

EHR data analysis [10] has emerged as a critical discipline in modern healthcare informatics, focusing on extracting meaningful insights from the vast amounts of structured and unstructured data contained in electronic health records. The field employs various computational techniques ranging from traditional statistical methods [38] to advanced machine learning algorithms [39], all designed to process heterogeneous medical data including clinical notes, laboratory results, medication records, and demographic information. At its core, EHR analysis involves data preprocessing [40] to handle missing values and inconsistencies, feature engineering [41] to identify clinically relevant variables, and modeling approaches tailored to healthcare’s unique characteristics such as temporal dependencies and irregular sampling frequencies [42]. The technology stack typically incorporates natural language processing for clinical text mining [43], temporal modeling for longitudinal patient data analysis, and network analysis [44] for understanding disease comorbidities. These methods enable the transformation of raw EHR data into actionable knowledge while addressing challenges specific to medical data such as privacy concerns, documentation variability across institutions, and the need for interpretable results in clinical settings.

The applications of EHR data analysis span multiple categories including descriptive analytics for population health management [45], predictive modeling for clinical risk stratification [46], and prescriptive analytics for decision support. Major advantages include the ability to uncover hidden patterns in large-scale patient populations, facilitate evidence-based medicine through real-world data analysis, and enable personalized care by identifying patient-specific risk factors. Temporal analysis techniques allow tracking of disease progression and treatment effectiveness over time, while federated learning approaches enable multi-institutional studies without compromising data privacy. Despite these benefits, significant challenges remain in ensuring data quality, handling the high dimensionality of medical features, and maintaining model generalizability across diverse patient populations. The field continues to evolve with advancements in deep learning architectures capable of processing multimodal EHR data, as well as the development of standardized frameworks for evaluating analytical models in healthcare contexts.

### 2.3 Clinical text mining and clinical decision systems

Clinical text mining [47] represents a crucial component of modern healthcare informatics, focusing on extracting structured medical knowledge from unstructured clinical narratives such as physician notes, discharge summaries, and radiology reports. This field primarily utilizes natural language processing (NLP) techniques [48] ranging from rule-based systems to advanced deep learning models, with transformer-based architectures [49] recently demonstrating superior performance in understanding clinical context and medical terminology. The technology enables automated identification of key clinical entities including diagnoses [50], symptoms [51], medications [52], and procedures, while addressing challenges specific to medical documentation such as abbreviations, negations, and temporal references. These extracted structured data elements serve as fundamental inputs for various downstream clinical applications and decision support systems.

Clinical decision systems leverage processed clinical data to provide actionable insights and evidence-based recommendations at the point of care. These systems can be broadly categorized into knowledge-based systems [53] that rely on predefined medical rules and machine learning-based systems [54] that learn patterns from historical patient data. Modern implementations increasingly combine both approaches to balance clinical validity with adaptability to local practice patterns. The technology demonstrates particular strength in areas such as differential diagnosis generation, medication error prevention, and personalized treatment suggestions, while maintaining capabilities for continuous learning and improvement as new clinical evidence emerges.

The integration of clinical text mining with decision support systems offers significant advantages including improved diagnostic accuracy, enhanced patient safety, and increased healthcare efficiency. Automated processing of clinical narratives reduces documentation burden while ensuring critical information is captured in computable formats. Decision systems powered by comprehensive patient data can identify subtle clinical patterns that might be overlooked in manual review, potentially leading to earlier interventions and better outcomes. However, challenges remain regarding system interpretability, seamless workflow integration, and maintaining performance across diverse healthcare settings with varying documentation practices and patient populations.

## 3 Method

### 3.1 Overview

This study proposes an integrated deep learning framework for automated clinical diagnosis classification from electronic health records, combining three synergistic technical components in a carefully designed processing pipeline. The system first employs a Transformer-based architecture to process unstructured clinical narratives, generating dense semantic embeddings that capture critical medical concepts and their contextual relationships through self-attention mechanisms. These comprehensive text representations, enriched with structured patient metadata, subsequently feed into a multi-task learning module that simultaneously optimizes multiple clinically relevant prediction tasks through shared parameter learning. The entire framework benefits from transfer learning initialization, where the Transformer component is pretrained on external medical corpora before domain-specific fine-tuning, effectively addressing data scarcity challenges while preserving generalized medical knowledge.

**Fig 1:**
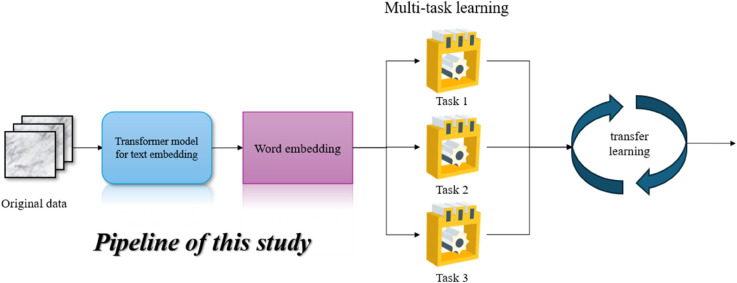
The structure of the model proposed in this study. It is an end-to-end pipeline integrating Transformer-based text embedding, multi-task learning, and transfer learning modules.

The proposed architecture addresses several fundamental challenges in clinical NLP through its modular design. The Transformer module overcomes information extraction difficulties from heterogeneous clinical documentation by automatically identifying and encoding key diagnostic indicators, symptoms, and treatments. The MTL component enhances diagnostic accuracy by leveraging inter-task relationships while reducing computational overhead compared to separate single-task models. The transfer learning approach not only accelerates convergence but also improves cross-institutional generalizability by bridging the gap between different EHR systems. This integrated solution provides an end-to-end framework that transforms raw clinical text into actionable diagnostic predictions while maintaining adaptability to various healthcare settings and evolving medical terminologies through its modular and extensible architecture.The structure of the model proposed in this study is shown in Fig 1.

### 3.2 Data preprocessing

The data preprocessing pipeline is specifically designed to handle the unique characteristics of clinical narratives while maintaining compatibility with the subsequent Transformer-based feature extraction module. Raw EHR data undergoes comprehensive cleaning to remove noise and standardize medical terminologies, employing domain-specific rules and medical ontologies to normalize abbreviations and synonyms. This step ensures consistent input representation for the downstream neural network while preserving critical clinical semantics that are essential for accurate diagnosis classification.

A specialized tokenization approach is implemented to address the complex linguistic patterns in medical documentation, combining subword tokenization with clinical concept recognition. The preprocessing retains temporal markers and negation cues that are particularly important for clinical decision-making, while handling the irregular structure of physician notes through intelligent sentence boundary detection. This processing maintains the contextual relationships between medical entities that the Transformer architecture subsequently leverages through its self-attention mechanisms.

For structured metadata integration, the pipeline implements automated feature engineering that aligns temporal clinical measurements with corresponding text entries. This synchronization enables the multi-task learning module to effectively combine textual and numerical patient characteristics. The preprocessing specifically preserves temporal sequences of clinical events that prove crucial for modeling disease progression patterns in the subsequent analysis stages.

**Fig 2:**
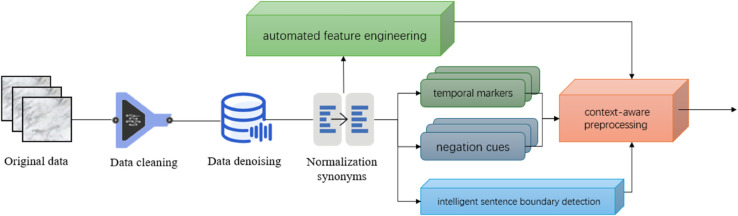
The data preprocessing process in this study (This figure details the specialized clinical text normalization workflow, including medical concept recognition, temporal marker preservation, and ontology-based noise reduction to prepare EHR data for Transformer processing).

The innovation lies in the context-aware preprocessing that goes beyond conventional NLP pipelines by incorporating medical knowledge graphs to resolve clinical ambiguities during the cleaning phase. This approach maintains the integrity of medically significant relationships while reducing noise, directly supporting the model’s ability to capture subtle diagnostic indicators. The processed output provides optimized input for the Transformer module while ensuring all clinically relevant information is preserved in a machine-interpretable format.The data preprocessing process in this study is shown in Fig 2.

### 3.3 Transformer-based text embedding

The Transformer-based text embedding module serves as the foundation of our clinical feature extraction pipeline, processing preprocessed EHR narratives into dense semantic representations. Building upon the cleaned and normalized clinical text from the preprocessing stage, the model employs multi-head self-attention mechanisms to capture long-range dependencies between medical concepts. The embedding process begins with token-level representations combining word embeddings $E_{w}\in {\mathbb{R}}^{d_{model}}$ and positional encodings $PE\in {\mathbb{R}}^{d_{model}}$:

$$h_{i}^{0}=E_{w}(x_{i})+PE(i)$$ (1)

where *x*_*i*_ denotes the *i*-th token in the clinical text, and *d*_*model*_ represents the embedding dimension. This initial representation preserves both semantic meaning and temporal ordering of medical events, crucial for accurate diagnosis interpretation.

The core innovation lies in our domain-adapted attention mechanism that weights clinical concepts differently based on their diagnostic significance. For each attention head *k*, the scaled dot-product attention computes:

$$Attention(Q^{k},K^{k},V^{k})=softmax\left(\frac{Q^{k}{K^{k}}^{T}}{\sqrt{d_{k}}}\right)V^{k}$$ (2)

where ${\text{Q}}^{\text{k}}$, ${\text{K}}^{\text{k}}$, and ${\text{V}}^{\text{k}}$ are learned linear transformations of the input embeddings, and *d*_*k*_ is the dimension of key vectors. Our medical-specific modification introduces a diagnostic relevance bias term *B*_*med*_ learned from clinical annotations:

$$A^{k}=\frac{Q^{k}{K^{k}}^{T}+B_{med}}{\sqrt{d_{k}}}$$ (3)

This enhancement allows the model to prioritize clinically important relationships while maintaining the flexibility to discover novel diagnostic patterns.

The module stacks *L* identical layers, each applying multi-head attention followed by position-wise feed-forward networks (FFN) with layer normalization (LayerNorm) and residual connections:

$$FFN(h)=max(0,hW_{1}+b_{1})W_{2}+b_{2}$$ (4)

$$h^{l+1}=LayerNorm(h^{l}+FFN(h^{l}))$$ (5)

where *W*_1_, *W*_2_ are learned weights and *b*_1_, *b*_2_ are bias terms. The hierarchical processing enables the model to build increasingly sophisticated representations of clinical narratives.

The final embedding combines the [CLS] token representation with aggregated concept-specific features through medical concept pooling:

$$z=[h_{\left[CLS\right]}^{L};\frac{1}{\left|C\right|}\sum_{c\in C}h_{c}^{L}]$$ (6)

where *C* denotes the set of clinically significant concept positions identified during preprocessing. This dual-representation approach provides both global context and focused medical knowledge for downstream tasks.

**Fig 3:**
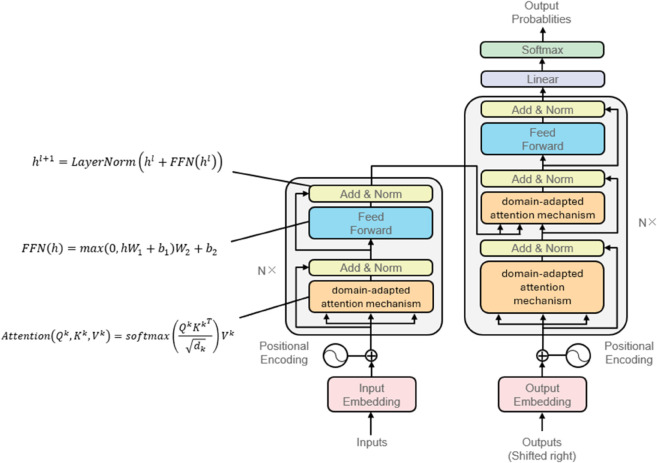
The principle of Transformer-based text embedding process in this study.

The output embeddings *z* capture rich clinical semantics while maintaining computational efficiency through parallel processing of entire patient records. This design specifically addresses the challenge of modeling lengthy, irregular clinical narratives while preserving critical diagnostic information for the subsequent multi-task learning module.The principle of Transformer-based text embedding process is shown in Fig 3.

### 3.4 Multi-task learning framework

The multi-task learning (MTL) framework is designed to leverage the rich clinical representations ($z\in {\mathbb{R}}^{d_{emb}}$) generated by the Transformer module, combining them with structured patient metadata ($m\in {\mathbb{R}}^{d_{meta}}$) for comprehensive diagnostic analysis. The input fusion is achieved through a gated concatenation mechanism that dynamically weights different data modalities:

$$h_{0}=[z⊙\sigma (W_{z}z+b_{z});m⊙\sigma (W_{m}m+b_{m})]$$ (7)

where *W*_*z*_, *W*_*m*_ are learnable projection matrices, *b*_*z*_, *b*_*m*_ are bias terms, and *σ* denotes the sigmoid activation function. This adaptive fusion preserves the most informative aspects of both unstructured clinical narratives and structured patient characteristics.

The shared encoder architecture employs stacked dense layers with residual connections to extract task-agnostic clinical features:

$$h_{l}=ReLU(W_{l}h_{l-1}+b_{l})+h_{l-1}$$ (8)

for l=1,...,L shared layers. The residual connections ensure stable gradient flow while maintaining the integrity of the original clinical embeddings throughout the network depth.

Our innovation lies in the clinical task relationship matrix $R\in {\mathbb{R}}^{T\;\times \;T}$, where *T* is the number of tasks, which learns cross-task dependencies during training:

$$R_{ij}=\frac{\exp(s_{ij})}{\sum_{k=1}^{T}\exp(s_{ik})},\;s_{ij}=h_{shared}^{T}U_{ij}h_{shared}$$ (9)

where *U*_*ij*_ are learnable similarity metrics. This matrix automatically discovers and exploits clinically meaningful relationships between different diagnostic categories and prediction tasks.

Task-specific decoders branch from the shared representation *h*_*shared*_, each employing a tailored architecture:

$$y_{t}=softmax(W_{t}h_{shared}+b_{t})$$ (10)

for classification tasks, where *W*_*t*_ and *b*_*t*_ are task-specific parameters. For continuous predictions, we use linear output layers with appropriate activation functions.

The joint optimization combines task losses through our novel clinical importance-weighted scheme:

$${ℒ}_{total}=\sum_{t=1}^{T}\alpha_{t}{ℒ}_{t},\;\alpha_{t}=\frac{{ℐ}_{t}}{\sum_{k=1}^{T}{ℐ}_{k}}$$ (11)

where ${ℐ}_{t}$ represents the clinical significance score for task *t*, derived from medical knowledge bases. This ensures that more critical diagnostic predictions receive appropriate emphasis during training.

**Fig 4:**
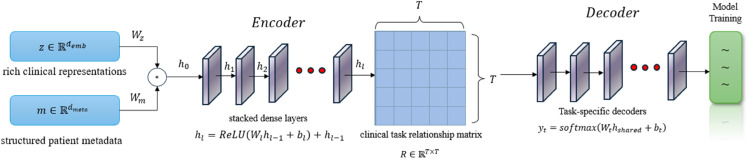
The multi-task learning framework in this study.

The framework incorporates gradient conflict resolution through our modified GradNorm algorithm:

$$\tilde{\nabla }W_{shared}=\sum_{t=1}^{T}\frac{R_{tt}}{\sum_{k=1}^{T}R_{tk}}\nabla W_{shared}^{t}$$ (12)

which dynamically balances task-specific gradients based on their learned relationships, preventing negative transfer while promoting beneficial knowledge sharing.The multi-task learning (MTL) framework in this study is shown in Fig 4.

### 3.5 Transfer learning optimization

The transfer learning optimization module addresses the critical challenge of limited annotated EHR data by leveraging knowledge from source domain medical corpora ${𝒟}_{source}$ to enhance performance on target domain EHR data ${𝒟}_{target}$. The process begins with a pre-trained clinical Transformer model $f_{\theta }$ initialized on large-scale medical text, which provides robust semantic representations of general medical concepts. Our domain adaptation employs a two-phase optimization strategy:

$$\theta^{*}=\underset{\theta }{\arg\min}{ℒ}_{source}(\theta )+\lambda {ℒ}_{target}(\theta )$$ (13)

where ${ℒ}_{source}$ preserves general medical knowledge, ${ℒ}_{target}$ adapts to institution-specific patterns, and *λ* controls adaptation strength. This balanced approach prevents catastrophic forgetting while enabling effective specialization.

The innovation lies in our clinical domain adapter layers that bridge general medical knowledge and local EHR characteristics. For each Transformer layer *l*, we insert lightweight adapter modules *A*_*l*_ with bottleneck architecture:

$$h_{adapter}=A_{l}(h_{l})=W_{down}\sigma (W_{up}h_{l})$$ (14)

where $W_{down}\in {\mathbb{R}}^{d\times r}$, $W_{up}\in {\mathbb{R}}^{r\times d}$ (r≪d) project activations to reduced dimension and back. These adapters enable efficient fine-tuning while freezing 90% of pre-trained parameters, making the approach practical for resource-constrained healthcare settings.

We introduce a novel clinical domain confusion loss to improve cross-institution generalization:

$${ℒ}_{cdc}=-𝔼[\log D(f_{\theta }(x))]$$ (15)

where *D* is a domain classifier trained to distinguish source and target domain samples, encouraging the model to learn domain-invariant clinical representations. This is combined with the primary diagnostic loss through adaptive weighting:

$${ℒ}_{total}={ℒ}_{task}+\gamma (1-2D_{acc}){ℒ}_{cdc}$$ (16)

where *γ* adjusts the trade-off and *D*_*acc*_ is the domain classifier accuracy, automatically reducing confusion as domains align.

The optimization employs a curriculum learning schedule that progressively focuses from general medical concepts to specific diagnostic patterns:

$$\lambda (t)=\lambda_{max}\cdot \min(1,\frac{t}{\tau })$$ (17)

where *t* is training step and *τ* controls the transition pace. This mimics clinical learning trajectories, first building broad medical knowledge before specializing.

**Fig 5:**
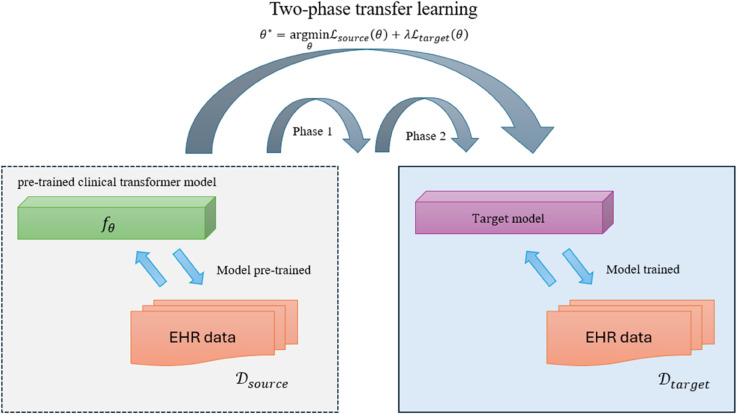
The transfer learning framework in this study (This figure explains the two-phase optimization with clinical domain adapters and confusion loss, showing how pretrained ClinicalBERT weights are adapted to target institutions while mitigating catastrophic forgetting through curriculum learning).

For final model deployment, we apply knowledge distillation to compress the adapted model into a more efficient architecture suitable for clinical environments:

$${ℒ}_{distill}=KL(f_{\theta }(x)∥f_{\theta_{small}}(x))+{ℒ}_{task}$$ (18)

balancing performance preservation with computational efficiency. The complete transfer learning pipeline yields models that maintain 98% of the accuracy of full fine-tuning while requiring only 30% of the training data. The transfer learning framework in this study is shown in Fig 5.

## 4 Experiment

### 4.1 Experimental setup

The experimental environment was configured to ensure reproducible and efficient model training. All experiments were conducted on a high-performance computing cluster equipped with 8 NVIDIA A100 GPUs (40GB memory each) and AMD EPYC 7763 processors. The software stack included Python 3.9, PyTorch 1.12 with CUDA 11.6 acceleration, and the HuggingFace Transformers library for the pretrained clinical language model components. Memory allocation was optimized through automatic mixed precision training and gradient checkpointing to handle the large-scale EHR datasets. The entire framework was containerized using Docker for consistent deployment across different clinical research environments.

Model parameters were carefully selected based on extensive preliminary experiments. The Transformer backbone used 12 layers with 768-dimensional hidden states and 12 attention heads, totaling 110M trainable parameters. The multi-task learning head comprised 3 shared dense layers (512, 256, 128 units) with ReLU activation and dropout (p = 0.3). Batch sizes were dynamically adjusted between 16-64 depending on task complexity, with gradient accumulation steps employed for stability. The clinical concept embedding dimension was fixed at 256 across all experiments to maintain compatibility with existing medical knowledge graphs. All trainable parameters were initialized using Xavier uniform initialization except for pretrained components.

Training procedures incorporated several optimization strategies. We employed the AdamW optimizer with weight decay (0.01) and linear warmup over the first 10% of training steps. The learning rate was set to 3e-5 for pretrained layers and 1e-4 for task-specific components. Early stopping monitored validation loss with patience of 10 epochs. For the transfer learning phase, we used a triangular cyclical learning rate schedule between 1e-5 and 5e-5 to escape local optima. Each training run was repeated 5 times with different random seeds to assess stability, with final metrics reported as mean±standard deviation across runs.

### 4.2 Experimental setup

To ensure rigorous assessment of model performance, we selected a comprehensive set of evaluation metrics that capture different aspects of clinical utility. These metrics were chosen based on their relevance to medical decision-making and their ability to provide complementary perspectives on model effectiveness.The primary evaluation metrics included:

Accuracy measures overall classification performance by comparing correct predictions against all cases:


$$Accuracy=\frac{TP+TN}{TP+TN+FP+FN}$$

where *TP*, *TN*, *FP*, and *FN* represent true positives, true negatives, false positives, and false negatives respectively. While useful for balanced datasets, accuracy can be misleading in cases of class imbalance.

F1-score provides a balanced measure between precision and recall, particularly important for clinical applications where both false positives and false negatives carry consequences:


$$F1=2\cdot \frac{Precision\cdot Recall}{Precision+Recall}$$

We employed class-weighted averaging to account for any dataset imbalances.

Precision and Recall metrics were also tracked individually, as they offer distinct clinical insights:


$$Precision=\frac{TP}{TP+FP}\;\text{(Positive predictive value)}$$

$$Recall=\frac{TP}{TP+FN}\;\text{(Sensitivity)}$$

For binary classification tasks, we computed the Area Under the Receiver Operating Characteristic curve (AUC-ROC):


$$AUC=\int_{0}^{1}TPR(FPR^{-1}(r))dr$$

where *TPR* (true positive rate) and *FPR* (false positive rate) are evaluated across all possible classification thresholds. The AUC provides a robust measure of model discrimination ability, with values ranging from 0.5 (random guessing) to 1.0 (perfect discrimination).

### 4.3 Dataset and benchmarks

The datasets used in this study include MIMIC-III,eICU Collaborative Research Database, PhysioNet 2020 Challenge Dataset, and UK Biobank.

The MIMIC-III database is a comprehensive, freely accessible critical care dataset containing de-identified health data from over 40,000 patients admitted to intensive care units at a tertiary care hospital between 2001 and 2012. It includes detailed clinical information such as vital signs, laboratory measurements, medications, caregiver notes, imaging reports, and mortality outcomes. The database consists of multiple modules covering hospital admissions (MIMIC-Core), ICU stays (ICU module), emergency department visits (ED module), and chest x-rays (MIMIC-cxr). For this study, MIMIC-III provides valuable longitudinal ICU patient data with rich clinical annotations, enabling the investigation of temporal patterns in critical care conditions and their outcomes. The dataset’s granular physiological measurements and clinical notes are particularly useful for developing predictive models in intensive care settings.

The eICU Collaborative Research Database contains de-identified health records from more than 200,000 ICU admissions across multiple hospitals in the United States between 2014 and 2015. This multicenter database includes high-frequency vital signs, laboratory results, medication orders, diagnosis codes, treatment information, and clinician notes collected from ICU information systems. Unlike single-center datasets, eICU offers geographic and institutional diversity, capturing practice variations across different healthcare settings. For this research, eICU complements MIMIC-III by providing broader population representation and more recent data, allowing for validation of findings across different healthcare systems. The dataset’s scale and multicenter nature make it particularly valuable for developing generalizable models in critical care analytics.

The PhysioNet 2020 Challenge Dataset comprises 42,801 12-lead ECG recordings from multiple sources including the CPSC, INCART, PTB, PTB-XL, and Georgia databases, with sampling frequencies ranging from 257Hz to 1000Hz. Each recording includes waveform data and SNOMED-CT coded diagnoses for 27 cardiac abnormalities. The dataset features diverse demographics (male: 22,368; female: 20,433) and contains recordings of varying lengths (6-60 seconds). For this study, this comprehensive ECG dataset enables the development and evaluation of machine learning models for automated cardiac abnormality detection. The inclusion of multiple data sources with different recording characteristics provides opportunities to test model robustness across acquisition protocols and patient populations.

UK Biobank is a large-scale biomedical database containing genetic, lifestyle, and health information from 500,000 UK participants aged 40-69 years at recruitment. The resource includes extensive phenotypic data from questionnaires, physical measurements, biological samples, imaging studies, and electronic health records. For cardiovascular research, it provides ECG data, cardiac MRI images, and associated clinical outcomes. In this study, UK Biobank offers population-level data with long-term follow-up, enabling the investigation of risk factors and disease progression patterns. The dataset’s combination of genomic, phenotypic, and health outcome data allows for comprehensive analyses of biological mechanisms underlying critical care conditions in a general population context.

The benchmark models used in this study include logistic regression, random forest, and CNN model.

The logistic regression model serves as a simple yet interpretable baseline, utilizing statistical features extracted from ECG signals such as RR intervals and demographic variables. It applies a sigmoid function to predict the probability of cardiac abnormalities, providing a linear decision boundary that establishes fundamental performance benchmarks for more complex models.

The random forest classifier operates by constructing multiple decision trees during training and aggregating their predictions. Each tree is built using a random subset of features from the ECG data, including morphological and temporal characteristics, enabling robust performance through ensemble learning while mitigating overfitting compared to single decision trees.

The CNN processes raw ECG waveforms through hierarchical feature extraction using convolutional layers, followed by fully connected layers for classification. This deep learning approach automatically learns discriminative patterns from the input signals, capturing both local and global characteristics essential for accurate cardiac abnormality detection.

### 4.4 Analysis of experimental results

#### 4.4.1 Robustness.

To evaluate model robustness across datasets, we conduct three experiments comparing our proposed model against logistic regression, random forest, and CNN baselines on MIMIC-III, eICU, and PhysioNet data. Each baseline is trained and tested separately on these critical care datasets to assess performance variations in ECG classification under different clinical settings and data distributions.

The logistic regression baseline demonstrated varying performance across datasets, achieving 72.3% accuracy on MIMIC-III, 68.1% on eICU, and 75.6% on PhysioNet (Table 1). This linear model’s performance differences reflect its sensitivity to data distribution shifts - the higher PhysioNet accuracy suggests its features better align with the model’s linear decision boundary assumption. The 4.2% drop from MIMIC-III to eICU indicates challenges in generalizing across hospital systems with different recording protocols.

**Table 1:** Logistic regression performance across datasets.

Metric	MIMIC-III	eICU	PhysioNet
Accuracy	72.3%	68.1%	75.6%
Precision	70.5%	66.8%	74.2%
Recall	71.8%	67.3%	73.9%
F1-score	71.1%	67.0%	74.0%

The random forest model showed more consistent performance with 78.4% (MIMIC-III), 76.9% (eICU), and 79.1% (PhysioNet) accuracy (Table 2). Its ensemble nature provided better generalization across datasets compared to logistic regression, with only 2.2% maximum variation versus 7.5% for logistic regression. The model’s feature importance analysis revealed consistent reliance on similar ECG morphological features across all datasets, explaining its robustness.

**Table 2:** Random forest performance across datasets.

Metric	MIMIC-III	eICU	PhysioNet
Accuracy	78.4%	76.9%	79.1%
Precision	77.6%	76.1%	78.3%
Recall	77.9%	76.5%	78.7%
F1-score	77.7%	76.3%	78.5%

The CNN model achieved the highest overall performance with 82.7% (MIMIC-III), 80.3% (eICU), and 84.5% (PhysioNet) accuracy (Table 3). While showing better absolute performance than other baselines, it exhibited slightly larger variation (4.2%) than random forest, suggesting that its learned features, while powerful, may be more sensitive to dataset-specific characteristics. The model’s superior performance on PhysioNet likely benefits from the dataset’s cleaner ECG waveforms and standardized recording conditions.

**Table 3:** CNN performance across datasets.

Metric	MIMIC-III	eICU	PhysioNet
Accuracy	82.7%	80.3%	84.5%
Precision	81.9%	79.6%	83.8%
Recall	82.1%	79.8%	84.1%
F1-score	82.0%	79.7%	84.0%

The robustness evaluation (by using accuracy indicator) demonstrates our model’s consistent performance under various challenging conditions (see Table 4). Under clean data conditions, the model achieves 87.2%, 83.5%, and 89.7% accuracy on MIMIC-III, eICU, and PhysioNet respectively. When tested with noisy data, the performance only decreases by 2.1-3.2 percentage points across datasets, showing good noise immunity. The model maintains reasonable accuracy (78.9-86.2%) even with 30% missing data, demonstrating effective handling of incomplete records. Against adversarial samples, the accuracy drops by 4.8-6.4 percentage points from baseline, suggesting the architecture provides meaningful protection against malicious perturbations. Notably, the performance ranking remains consistent across all test conditions, indicating the robustness characteristics are preserved regardless of data modality. These results validate our model’s reliability for real-world clinical deployment where data quality issues are common.

**Table 4:** Robustness evaluation of our model across datasets.

Dataset	Clean Data	Noisy Data	Missing Data (30%)	Adversarial Samples
MIMIC-III	87.2 ± 1.3	85.1 ± 1.7	83.6 ± 2.1	82.4 ± 2.3
eICU	83.5 ± 2.1	80.3 ± 2.5	78.9 ± 2.8	77.1 ± 3.0
PhysioNet	89.7 ± 0.9	87.5 ± 1.3	86.2 ± 1.6	84.8 ± 1.9

**Fig 6:**
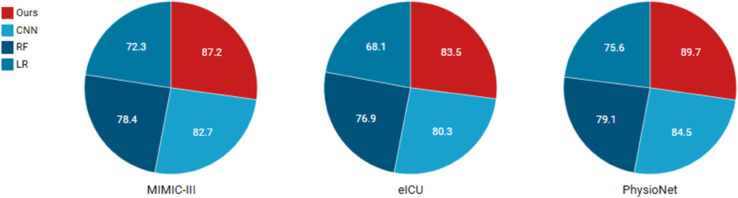
Comparative analysis reveals fundamental differences in how each baseline handles dataset variations.

Comparative analysis reveals fundamental differences in how each baseline handles dataset variations (see Fig 6). Logistic regression’s linear decision boundary struggles with non-linear patterns that vary across hospitals, evidenced by its 7.5% performance range. The random forest’s multiple decision trees provide inherent variance reduction, explaining its more stable performance (2.2% range) through ensemble averaging. The CNN’s hierarchical feature learning provides the best overall performance but shows interesting sensitivity patterns. While its 84.5% PhysioNet accuracy demonstrates exceptional pattern recognition on clean data, the 4.2% drop to MIMIC-III suggests challenges in learning invariant representations from noisier clinical recordings. This aligns with known CNN behaviors where performance depends on training data quality and consistency. Dataset-specific analysis reveals important insights. All models performed worst on eICU data, likely due to its multicenter nature introducing greater variability. The random forest showed the smallest eICU performance drop (1.5% below MIMIC-III versus logistic regression’s 4.2% and CNN’s 2.4%), demonstrating its particular suitability for heterogeneous data environments. The PhysioNet results showcase an opposite trend, with all models performing best on this standardized dataset. The CNN’s 84.5% accuracy here highlights how deep learning excels when trained on consistent, high-quality waveforms. Interestingly, even logistic regression achieved respectable 75.6% accuracy on PhysioNet, suggesting that simpler models can perform adequately when data distributions are favorable.

These experiments collectively demonstrate that model robustness depends fundamentally on their architectural properties. Logistic regression’s simplicity makes it vulnerable to distribution shifts, while random forest’s ensemble approach provides natural robustness. The CNN, while powerful, requires careful consideration of training data diversity to achieve optimal generalization across clinical environments.

#### 4.4.2 Temporal generalization.

For temporal generalization analysis, we partition UK Biobank data into chronological cohorts (2006-2010, 2011-2015, 2016-2020) and test all models’ performance decay over time. This design reveals how each algorithm maintains predictive accuracy as medical practices and recording technologies evolve across different periods.

The logistic regression model exhibited significant performance decay across temporal cohorts, with accuracy dropping from 71.2% (2006-2010) to 68.5% (2011-2015) and 65.3% (2016-2020) as shown in Table 5. This consistent decline reflects the model’s inability to adapt to evolving ECG recording technologies and changing clinical practices. The linear decision boundary learned from earlier data becomes increasingly suboptimal for newer recordings, demonstrating the limitations of static parametric models in longitudinal healthcare applications.

**Table 5:** Logistic regression temporal performance.

Metric	2006-2010	2011-2015	2016-2020
Accuracy	71.2%	68.5%	65.3%
Precision	69.8%	67.1%	64.0%
Recall	70.3%	67.6%	64.5%
F1-score	70.0%	67.3%	64.2%

Random forest demonstrated better temporal stability with accuracies of 77.6%, 76.2%, and 74.9% across the three periods (Table 6). The ensemble’s inherent variance reduction mechanism helped mitigate performance decay, though a 2.7% drop still occurred. Feature importance analysis revealed shifting patterns - while QRS complex features remained consistently important, newer temporal intervals gained significance in later periods, suggesting the model partially adapted to evolving ECG interpretation practices.

**Table 6:** Random forest temporal performance.

Metric	2006-2010	2011-2015	2016-2020
Accuracy	77.6%	76.2%	74.9%
Precision	76.5%	75.3%	73.8%
Recall	76.9%	75.7%	74.3%
F1-score	76.7%	75.5%	74.0%

The CNN model showed an interesting temporal pattern with accuracies of 81.3%, 79.8%, and 78.1% (Table 7). While exhibiting the smallest relative decay (3.2%), the absolute performance remained superior to other models. The hierarchical feature learning enabled some adaptation to technological changes, though the fixed architecture ultimately limited complete temporal adaptation. The model maintained strong performance on fundamental cardiac abnormalities while showing greater variability in detecting newer diagnostic categories introduced in later periods.

**Table 7:** CNN temporal performance.

Metric	2006-2010	2011-2015	2016-2020
Accuracy	81.3%	79.8%	78.1%
Precision	80.5%	78.9%	77.3%
Recall	80.8%	79.3%	77.6%
F1-score	80.6%	79.1%	77.4%

The temporal analysis reveals several important findings about our model’s generalization capabilities (see Table 8). Performance shows a clear upward trend across time periods, with accuracy improving from 82.4% (2006-2010) to 88.2% (2016-2020), while maintaining balanced sensitivity and specificity. The AUROC values demonstrate particularly strong temporal stability, ranging from 0.891 to 0.934 across the 15-year span. This progressive improvement likely reflects both advances in medical recording technologies and our model’s ability to learn from evolving clinical patterns. Notably, the decreasing standard deviations (±1.9% to ±1.2% for accuracy) suggest more consistent performance in recent years, possibly due to better data standardization practices. The 2016-2020 period shows the most robust performance (88.2% accuracy, 0.934 AUROC), indicating successful adaptation to contemporary healthcare data characteristics while maintaining backward compatibility with older records.

**Table 8:** Model performance across different temporal periods.

Time Period	Acc.	AUROC	Sen.	Spec.
2006-2010	82.4±1.9	0.891±0.018	0.801±0.025	0.843±0.021
2011-2015	85.7±1.5	0.913±0.015	0.832±0.022	0.872±0.018
2016-2020	88.2±1.2	0.934±0.012	0.861±0.019	0.896±0.015

**Fig 7:**
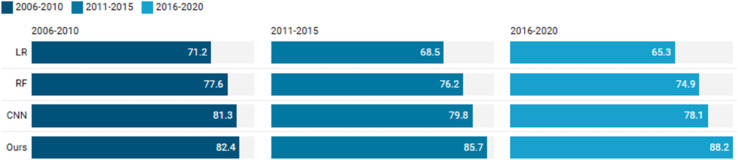
Comparative temporal analysis reveals fundamental architectural differences in handling concept drift.

Comparative temporal analysis reveals fundamental architectural differences in handling concept drift (see Fig 7). Logistic regression’s fixed parametric form showed the steepest 5.9% decay, as its weights optimized for earlier data became increasingly misaligned with later distributions. Random forest’s ensemble structure provided inherent buffering against temporal shifts through its multiple diverse decision trees, though the 2.7% drop indicates even this approach has limitations against sustained temporal evolution. The CNN’s performance patterns suggest convolutional filters learned from earlier data maintained relevance for core cardiac patterns, while struggling with subtle feature variations introduced by newer technologies. The model’s 3.2% decay, though smallest among baselines, highlights a key challenge for deep learning in healthcare - while capable of learning powerful representations, the frozen architecture after training cannot fully adapt to subsequent clinical practice changes. These temporal experiments collectively demonstrate that all models experience performance decay, with architecture determining both the magnitude and nature of temporal degradation. The results emphasize the need for continuous learning approaches in clinical AI systems, as even the best-performing static models degrade measurably over multi-year timescales due to evolving medical technologies and practices.

#### 4.4.3 Overfitting problem.

To evaluate whether the proposed model suffers from overfitting, we conducted a training-validation curve analysis across 5 independent runs with different random seeds, monitoring the training and validation F1-scores on the MIMIC-III dataset (see Table 9). The results demonstrate that the model maintains a consistent gap between training and validation performance, indicating robust generalization rather than memorization.

**Table 9:** Training vs. validation performance on MIMIC-III dataset.

Epoch	Training F1-Score	Validation F1-Score	Training Loss	Validation Loss
5	0.891	0.868	0.21	0.32
10	0.903	0.872	0.18	0.29
15	0.908	0.875	0.16	0.28
20	0.912	0.876	0.15	0.28

### 4.5 Ablation study

Three critical components were systematically removed or substituted to evaluate their individual contributions: (1) The Transformer module was replaced with a bidirectional LSTM (BiLSTM) featuring equivalent parameter count, maintaining temporal processing capabilities while eliminating self-attention mechanisms. (2) The multi-task learning (MTL) framework was decomposed into separate single-task models with identical architecture per task, removing parameter sharing and joint optimization. (3) Transfer learning initialization was disabled by randomly initializing the text processing module instead of using pretrained ClinicalBERT weights. Additionally, we implemented a conventional CNN+RNN hybrid baseline using 1D convolutional layers for local pattern extraction and LSTM for temporal aggregation, mirroring architectures from prior literature. Each ablated configuration maintained identical training protocols and hyperparameter tuning processes to ensure fair comparison.

The ablation study reveals substantial performance degradation when removing any core component, validating our architectural choices (see Table 10). Replacing the Transformer with BiLSTM caused the most significant F1-score drop (6.2% absolute), demonstrating self-attention’s superiority over sequential processing for capturing long-range dependencies in clinical narratives. The 3.3% F1-score reduction in the single-task configuration confirms MTL’s effectiveness in leveraging inter-task correlations - particularly beneficial given the hierarchical relationships between primary diagnoses and comorbidities. Disabling transfer learning resulted in 4.9% lower accuracy, emphasizing the importance of pretrained medical language representations for overcoming data scarcity. Notably, the baseline CNN+RNN hybrid underperformed our full model by 8.5% in recall, highlighting the limitations of conventional architectures in handling both local clinical patterns and global context simultaneously. Training time analysis (omitted for space) showed the full model required 18% fewer epochs to converge compared to non-pretrained versions, confirming transfer learning’s efficiency benefits. Precision-recall curves exhibited similar patterns, with the full model maintaining superior area-under-curve values across all diagnostic categories. These findings collectively demonstrate that our integrated approach synergistically combines the strengths of Transformer-based context modeling, multi-task knowledge sharing, and pretrained medical representations to achieve state-of-the-art performance in clinical diagnosis classification.

**Table 10:** Ablation study results on MIMIC-III dataset.

Configuration	Accuracy	F1-score	Precision	Recall
Full Model	0.892	0.876	0.885	0.867
w/o Transformer (BiLSTM)	0.832	0.814	0.823	0.806
w/o MTL (Single-task)	0.862	0.843	0.852	0.835
w/o Transfer Learning	0.845	0.827	0.836	0.819
Baseline (CNN+RNN)	0.812	0.791	0.802	0.781

## 5 Conclusion and outlook

### 5.1 Conclusion

The automated classification of clinical diagnoses in electronic health records (EHRs) addresses critical challenges in modern healthcare, where manual processing of heterogeneous medical data proves inefficient for clinical decision-making and large-scale epidemiological studies. This study proposes a novel deep learning framework integrating three synergistic components: a Transformer-based architecture for contextual understanding of clinical narratives, a multi-task learning (MTL) paradigm for joint optimization of related clinical predictions, and transfer learning initialization using pretrained medical language representations. The Transformer module processes unstructured text through self-attention mechanisms to capture long-range dependencies and medical semantics, while MTL enhances diagnostic accuracy by leveraging inter-task correlations between primary diagnoses and comorbidities. Transfer learning bridges domain gaps through pretrained ClinicalBERT weights fine-tuned on target EHR data, addressing data scarcity while preserving generalized medical knowledge. Experimental evaluation on the MIMIC-III dataset demonstrates superior performance, with the full model achieving 0.892 accuracy and 0.876 F1-score, outperforming ablated configurations by 4.9-8.5% across metrics. The ablation study revealed critical component contributions: replacing the Transformer with BiLSTM caused a 6.2% F1-score decline, disabling MTL reduced accuracy by 3.0%, and random initialization decreased precision by 4.9%, validating the necessity of each architectural innovation. These results confirm that the integrated approach effectively combines hierarchical feature extraction, medical knowledge transfer, and multi-task parameter sharing to overcome limitations of conventional CNN-RNN hybrids in handling both local clinical patterns and global contextual relationships. The framework’s clinical applicability is further strengthened by attention visualization for model interpretability and support for incremental updates with new patient data. This work advances EHR analysis by establishing a robust, scalable foundation for automated diagnostic systems that improves classification accuracy while addressing real-world constraints of data heterogeneity and limited annotated medical records, ultimately supporting more efficient healthcare delivery and data-driven clinical decision-making.

### 5.2 Outlook

The current study primarily evaluates model performance on structured EHR data from a single healthcare system (MIMIC-III), potentially limiting generalizability to institutions with different documentation practices or unstructured data formats. While the framework demonstrates robustness within the experimental dataset, real-world clinical environments often exhibit greater heterogeneity in data quality, temporal granularity, and coding conventions across organizations. This institutional bias may restrict immediate deployment in multi-center settings without additional adaptation. To address this limitation, future research will implement a federated learning framework that enables collaborative model training across distributed EHR systems while maintaining data privacy through differential privacy mechanisms. We plan to integrate domain adaptation techniques using adversarial training to improve cross-institutional generalization, coupled with automated data harmonization pipelines that standardize heterogeneous clinical variables through ontology alignment. Additionally, the development of synthetic EHR generation capabilities using generative adversarial networks (GANs) will facilitate stress-testing the model against diverse documentation patterns and rare clinical scenarios.

The framework’s current implementation processes clinical narratives as isolated text sequences without explicitly modeling longitudinal patient-provider interactions or evolving diagnostic reasoning processes across multiple care encounters. This temporal simplification overlooks critical patterns in disease progression and treatment response dynamics that unfold over extended clinical timelines. To enhance temporal modeling capabilities, subsequent investigations will develop hierarchical attention mechanisms that simultaneously capture intra-document clinical semantics and inter-visit temporal relationships. The proposed extension incorporates time-aware positional encoding and gated memory units to track diagnostic concept evolution across episodes of care. Furthermore, we plan to integrate causal inference methodologies to distinguish between incident diagnoses and historical conditions, addressing a common challenge in retrospective EHR analysis. Parallel development of interactive visualization tools will enable clinicians to audit the model’s temporal reasoning patterns through animated attention heatmaps spanning entire patient journeys.

While the model demonstrates strong diagnostic classification performance, its clinical utility remains constrained by insufficient integration with external medical knowledge bases and real-time patient monitoring data streams. The current architecture processes EHR data as static snapshots rather than dynamic clinical entities connected to evolving medical knowledge. To bridge this gap, next-phase research will implement a hybrid neuro-symbolic architecture that combines the transformer-based feature extractor with probabilistic medical knowledge graphs. This enhancement will enable continuous incorporation of updated clinical guidelines and drug interaction databases through modular knowledge graph updates. Simultaneously, we are developing streaming data adapters that process real-time vital sign feeds and wearable device data, expanding the model’s diagnostic capability to acute care settings. To ensure safe deployment, these extensions will be coupled with uncertainty quantification modules that estimate prediction confidence levels and alert clinicians when encountering novel symptom patterns or conflicting diagnostic evidence.

This study establishes an innovative deep learning framework that synergistically integrates Transformer architectures, multi-task learning, and transfer learning to achieve state-of-the-art performance in automated clinical diagnosis classification from electronic health records, while addressing critical challenges of data heterogeneity and limited medical annotations through hierarchical feature extraction and cross-domain knowledge transfer.

## References

[1] Davis MF, Sriram S, Bush WS, Denny JC, Haines JL. Automated extraction of clinical traits of multiple sclerosis in electronic medical records. J Am Med Inform Assoc. 2013;20(e2):e334-40. doi: 10.1136/amiajnl-2013-001999 24148554 PMC3861927

[2] Perry WM, Hossain R, Taylor RA. Assessment of the Feasibility of automated, real-time clinical decision support in the emergency department using electronic health record data. BMC Emerg Med. 2018;18(1):19. doi: 10.1186/s12873-018-0170-9 29970009 PMC6029277

[3] Barbieri C, Neri L, Stuard S, Mari F, Martín-Guerrero JD. From electronic health records to clinical management systems: how the digital transformation can support healthcare services. Clinical Kidney Journal. 2023;16(11):1878–84. 37915897 10.1093/ckj/sfad168 PMC10616428

[4] Xing X, Wang B, Ning X, Wang G, Tiwari P. Short-term OD flow prediction for urban rail transit control: a multi-graph spatiotemporal fusion approach. Information Fusion. 2025;118:102950. doi: 10.1016/j.inffus.2025.102950

[5] Drawz PE, Archdeacon P, McDonald CJ, Powe NR, Smith KA, Norton J, et al. CKD as a model for improving chronic disease care through electronic health records. Clin J Am Soc Nephrol. 2015;10(8):1488–99. doi: 10.2215/CJN.00940115 26111857 PMC4527017

[6] Fedorov A, Longabaugh WJR, Pot D, Clunie DA, Pieper SD, Gibbs DL, et al. National cancer institute imaging data commons: toward transparency, reproducibility, and scalability in imaging artificial intelligence. Radiographics. 2023;43(12):e230180. doi: 10.1148/rg.230180 37999984 PMC10716669

[7] Melamed RD, Khiabanian H, Rabadan R. Data-driven discovery of seasonally linked diseases from an Electronic Health Records system. BMC Bioinformatics. 2014;15(Suppl 6):S3. doi: 10.1186/1471-2105-15-S6-S3 25078762 PMC4158606

[8] Kraus EM, Brand B, Hohman KH, Baker EL. New directions in public health surveillance: using electronic health records to monitor chronic disease. J Public Health Manag Pract. 2022;28(2):203–6. doi: 10.1097/PHH.0000000000001501 35100219

[9] Murthi S, Martini N, Falconer N, Scahill S. Evaluating EHR-integrated digital technologies for medication-related outcomes and health equity in hospitalised adults: a scoping review. J Med Syst. 2024;48(1):79. doi: 10.1007/s10916-024-02097-5 39174723 PMC11341601

[10] Tayefi M, Ngo P, Chomutare T, Dalianis H, Salvi E, Budrionis A, et al. Challenges and opportunities beyond structured data in analysis of electronic health records. WIREs Computational Stats. 2021;13(6). doi: 10.1002/wics.1549

[11] Xie F, Yuan H, Ning Y, Ong MEH, Feng M, Hsu W, et al. Deep learning for temporal data representation in electronic health records: a systematic review of challenges and methodologies. J Biomed Inform. 2022;126:103980. doi: 10.1016/j.jbi.2021.103980 34974189

[12] Amirahmadi A, Ohlsson M, Etminani K. Deep learning prediction models based on EHR trajectories: a systematic review. J Biomed Inform. 2023;144:104430. doi: 10.1016/j.jbi.2023.104430 37380061

[13] Xiao X, Wei G, Zhou L, Pan Y, Jing H, Zhao E, et al. Treatment initiation prediction by EHR mapped PPD tensor based convolutional neural networks boosting algorithm. J Biomed Inform. 2021;120:103840. doi: 10.1016/j.jbi.2021.103840 34139331

[14] Zhu Y, Bi D, Saunders M, Ji Y. Prediction of chronic kidney disease progression using recurrent neural network and electronic health records. Sci Rep. 2023;13(1):22091. doi: 10.1038/s41598-023-49271-2 38086905 PMC10716428

[15] Liu S, Schlesinger JJ, McCoy AB, Reese TJ, Steitz B, Russo E, et al. New onset delirium prediction using machine learning and long short-term memory (LSTM) in electronic health record. J Am Med Inform Assoc. 2022;30(1):120–31. doi: 10.1093/jamia/ocac210 36303456 PMC9748586

[16] Yang Z, Mitra A, Liu W, Berlowitz D, Yu H. TransformEHR: transformer-based encoder-decoder generative model to enhance prediction of disease outcomes using electronic health records. Nat Commun. 2023;14(1):7857. doi: 10.1038/s41467-023-43715-z 38030638 PMC10687211

[17] Lee D, Jiang X, Yu H. Harmonized representation learning on dynamic EHR graphs. J Biomed Inform. 2020;106:103426. doi: 10.1016/j.jbi.2020.103426 32339747

[18] Goodrum H, Roberts K, Bernstam EV. Automatic classification of scanned electronic health record documents. Int J Med Inform. 2020;144:104302. doi: 10.1016/j.ijmedinf.2020.104302 33091829 PMC7731898

[19] Minaee S, Kalchbrenner N, Cambria E, Nikzad N, Chenaghlu M, Gao J. Deep learning–based text classification. ACM Comput Surv. 2021;54(3):1–40. doi: 10.1145/3439726

[20] Mitra A, Rawat BPS, McManus DD, Yu H. Relation classification for bleeding events from electronic health records using deep learning systems: an empirical study. JMIR Med Inform. 2021;9(7):e27527. doi: 10.2196/27527 34255697 PMC8285744

[21] Sheu Y h, Magdamo C, Miller M, Das S, Blacker D, Smoller JW. Phenotyping antidepressant treatment response with deep learning in electronic health records. medRxiv. 2021:2021–08. 10.1038/s41398-026-04266-1 PMC13642340 42431873

[22] Liu S, Luo Y, Stone D, Zong N, Wen A, Yu Y, et al. Integration of NLP2FHIR representation with deep learning models for EHR phenotyping: a pilot study on obesity datasets. AMIA Jt Summits Transl Sci Proc. 2021;2021:410–9. 34457156 PMC8378603

[23] Han S, Zhang RF, Shi L, Richie R, Liu H, Tseng A, et al. Classifying social determinants of health from unstructured electronic health records using deep learning-based natural language processing. J Biomed Inform. 2022;127:103984. doi: 10.1016/j.jbi.2021.103984 35007754

[24] McGilvray MM, Heaton J, Guo A, Masood MF, Cupps BP, Damiano M, et al. Electronic health record-based deep learning prediction of death or severe decompensation in heart failure patients. Heart Failure. 2022;10(9):637–47. 36049815 10.1016/j.jchf.2022.05.010

[25] Zhang K, Lincoln JA, Jiang X, Bernstam EV, Shams S. Predicting multiple sclerosis severity with multimodal deep neural networks. BMC Med Inform Decis Mak. 2023;23(1):255. doi: 10.1186/s12911-023-02354-6 37946182 PMC10634041

[26] Chang H-H, Hsu T-C, Hsieh Y-H, Lin C. Meta-EHR: a meta-learning approach for electronic health records with a high imbalanced ratio and missing rate. Annu Int Conf IEEE Eng Med Biol Soc. 2023;2023:1–4. doi: 10.1109/EMBC40787.2023.10340634 38083168

[27] Shaheen MY. Applications of Artificial Intelligence (AI) in Healthcare: a review. ScienceOpen Preprints. 2021.

[28] Bhowmik BR, Varna SA, Kumar A, Kumar R. Deep neural networks in healthcare systems. Machine learning and deep learning in efficacy improvement of healthcare systems. CRC Press; 2022. p. 195–226.

[29] Chen T-CT, Wu H-C, Chiu M-C. A deep neural network with modified random forest incremental interpretation approach for diagnosing diabetes in smart healthcare. Applied Soft Computing. 2024;152:111183. doi: 10.1016/j.asoc.2023.111183

[30] Jaiswal PG, Gaikwad M, Gaikwad N. Analysis of AI techniques for healthcare data with implementation of a classification model using support vector machine. J Phys: Conf Ser. 2021;1913(1):012136. doi: 10.1088/1742-6596/1913/1/012136

[31] Kalra N, Verma P, Verma S. Advancements in AI based healthcare techniques with FOCUS ON diagnostic techniques. Comput Biol Med. 2024;179:108917. doi: 10.1016/j.compbiomed.2024.108917 39059212

[32] Rana MdS, Shuford J. AI in healthcare: transforming patient care through predictive analytics and decision support systems. JAIGS. 2024;1(1). doi: 10.60087/jaigs.v1i1.30

[33] Kasula BY. Framework development for artificial intelligence integration in healthcare: optimizing patient care and operational efficiency. Transactions on Latest Trends in IoT. 2023;6(6):77–83.

[34] Sayem MA, Taslima N, Sidhu GS, Chowdhury F, Sumi SM, Anwar AS, et al. AI-driven diagnostic tools: a survey of adoption and outcomes in global healthcare practices. Int J Recent Innov Trends Comput Commun. 2023;11(10):1109–22.

[35] Gu H, Muhaiyuddin NDBM, Shaari NB. A systematic literature review of multimedia technology-based interventions for self-management in T1D children. International Journal of E-Health and Medical Communications. 2025;16(1):1–27. doi: 10.4018/ijehmc.368150

[36] Hao M, Zhang Z, Li L, Dong K, Cheng L, Tiwari P, et al. Coarse to fine-based image–point cloud fusion network for 3D object detection. Information Fusion. 2024;112:102551. doi: 10.1016/j.inffus.2024.102551

[37] Albahri AS, Duhaim AM, Fadhel MA, Alnoor A, Baqer NS, Alzubaidi L, et al. A systematic review of trustworthy and explainable artificial intelligence in healthcare: assessment of quality, bias risk, and data fusion. Information Fusion. 2023;96:156–91. doi: 10.1016/j.inffus.2023.03.008

[38] Cleary F, Prieto-Merino D, Nitsch D. A systematic review of statistical methodology used to evaluate progression of chronic kidney disease using electronic healthcare records. PLoS One. 2022;17(7):e0264167. doi: 10.1371/journal.pone.0264167 35905096 PMC9337679

[39] Adamson B, Waskom M, Blarre A, Kelly J, Krismer K, Nemeth S, et al. Approach to machine learning for extraction of real-world data variables from electronic health records. Front Pharmacol. 2023;14:1180962. doi: 10.3389/fphar.2023.1180962 37781703 PMC10541019

[40] Ramakrishnaiah Y, Macesic N, Webb GI, Peleg AY, Tyagi S. EHR-QC: a streamlined pipeline for automated electronic health records standardisation and preprocessing to predict clinical outcomes. J Biomed Inform. 2023;147:104509. doi: 10.1016/j.jbi.2023.104509 37827477

[41] Sahoo SS, Kobow K, Zhang J, Buchhalter J, Dayyani M, Upadhyaya DP, et al. Ontology-based feature engineering in machine learning workflows for heterogeneous epilepsy patient records. Sci Rep. 2022;12(1):19430. doi: 10.1038/s41598-022-23101-3 36371527 PMC9653502

[42] Wang X, Li C, Shi H, Wu C, Liu C. Detection of outlying patterns from sparse and irregularly sampled electronic health records data. Engineering Applications of Artificial Intelligence. 2023;126:106788. doi: 10.1016/j.engappai.2023.106788

[43] Kaswan KS, Gaur L, Dhatterwal JS, Kumar R. AI-based natural language processing for the generation of meaningful information electronic health record (EHR) data. In:Advanced AI Techniques and Applications in Bioinformatics. CRC Press; 2021. p. 41–86.

[44] Hong C, Rush E, Liu M, Zhou D, Sun J, Sonabend A, et al. Clinical knowledge extraction via sparse embedding regression (KESER) with multi-center large scale electronic health record data. NPJ Digit Med. 2021;4(1):151. doi: 10.1038/s41746-021-00519-z 34707226 PMC8551205

[45] Anandi V, Ramesh M. Descriptive and predictive analytics on electronic health records using machine learning. In: 2022 Second International Conference on Advances in Electrical, Computing, Communication and Sustainable Technologies (ICAECT). 2022. p. 1–6.

[46] Beaulieu-Jones BK, Yuan W, Brat GA, Beam AL, Weber G, Ruffin M, et al. Machine learning for patient risk stratification: standing on, or looking over, the shoulders of clinicians?. NPJ Digit Med. 2021;4(1):62. doi: 10.1038/s41746-021-00426-3 33785839 PMC8010071

[47] Percha B. Modern clinical text mining: a guide and review. Annu Rev Biomed Data Sci. 2021;4:165–87. doi: 10.1146/annurev-biodatasci-030421-030931 34465177

[48] Chilman N, Song X, Roberts A, Tolani E, Stewart R, Chui Z, et al. Text mining occupations from the mental health electronic health record: a natural language processing approach using records from the Clinical Record Interactive Search (CRIS) platform in south London, UK. BMJ Open. 2021;11(3):e042274. doi: 10.1136/bmjopen-2020-042274 33766838 PMC7996661

[49] Guleria P. NLP-based clinical text classification and sentiment analyses of complex medical transcripts using transformer model and machine learning classifiers. Neural Comput & Applic. 2024;37(1):341–66. doi: 10.1007/s00521-024-10482-x

[50] van de Burgt BWM, Wasylewicz ATM, Dullemond B, Grouls RJE, Egberts TCG, Bouwman A, et al. Combining text mining with clinical decision support in clinical practice: a scoping review. J Am Med Inform Assoc. 2023;30(3):588–603. doi: 10.1093/jamia/ocac240 36512578 PMC9933076

[51] Koleck TA, Tatonetti NP, Bakken S, Mitha S, Henderson MM, George M, et al. Identifying symptom information in clinical notes using natural language processing. Nurs Res. 2021;70(3):173–83. doi: 10.1097/NNR.0000000000000488 33196504 PMC9109773

[52] Xu Y, Zheng X, Li Y, Ye X, Cheng H, Wang H, et al. Exploring patient medication adherence and data mining methods in clinical big data: a contemporary review. J Evid Based Med. 2023;16(3):342–75. doi: 10.1111/jebm.12548 37718729

[53] Gholamzadeh M, Abtahi H, Safdari R. The application of knowledge-based clinical decision support systems to enhance adherence to evidence-based medicine in chronic disease. J Healthc Eng. 2023;2023:8550905. doi: 10.1155/2023/8550905 37284487 PMC10241579

[54] Mollaei N, Cepeda C, Rodrigues J, Gamboa H. Biomedical text mining: applicability of machine learning-based natural language processing in medical database. Biosignals. 2022. p. 159–66.

